# Investigating the Cellular Activity and Differential Gene Expression of Human Astrocytes in Interaction with Protein Composite Nanofibers

**DOI:** 10.1007/s11064-026-04855-y

**Published:** 2026-08-14

**Authors:** Li Yao, Karen Bustamante-Fuchs, Kayla Cantu, Teresa Shippy

**Affiliations:** 1 Department of Biological Sciences, Wichita State University, 1845 Fairmount Street, Wichita, KS 67260 USA; 2 Division of Biology, K-INBRE Data Science Core, Kansas State University, Manhattan, KS 66506 USA

**Keywords:** Astrocyte, Nanofiber, Collagen, Soy protein, Migration, Signaling pathway

## Abstract

Collagen is a major extracellular matrix component, and soy protein has been reported to influence cellular and immune-related processes. Nanofiber scaffolds incorporating collagen and soy protein isolate (SPI) may provide a platform for modulating cell–material interactions in neural systems. In this study, we fabricated electrospun nanofibers composed of collagen (CO), SPI, and polycaprolactone (PCL) and investigated the cellular and transcriptional responses of human astrocytes to these scaffolds in vitro. The nanofibers were characterized by scanning electron microscopy (SEM), Fourier transform infrared (FTIR) spectroscopy, and contact angle analysis. Human fetal astrocytes exhibited high viability on all nanofiber scaffolds. Flow cytometry analysis indicated that incorporation of SPI into CO/PCL nanofibers did not alter cell cycle distribution. Aligned nanofibers provided directional guidance for astrocyte migration. RNA-sequencing analysis revealed enrichment of the “neurodegeneration” and “antigen processing and presentation” pathways among the down-regulated genes in cells on CO/SPI/PCL fibers compared with CO/PCL fibers. Down-regulated genes in these pathways include IL1B, IL6, HLA-B, HLA-DMB, HLA-DPA1, and HLA-DRA. The “focal adhesion” pathway is enriched among up-regulated genes, which include COL4A1, COL4A2, FN1, LAMB1, LAMB2, AKT2, RAC1, RAC2, ROCK2, and PIP5K1A. These results demonstrate that incorporation of SPI into collagen-based nanofibers modulates astrocyte migration and gene expression profiles associated with focal adhesion and immune-related pathways, providing a foundation for further investigation of SPI-containing biomaterials in neural tissue engineering applications.

**Supplementary Information:**

The online version contains supplementary material available at 10.1007/s11064-026-04855-y.

## Introduction

Nanofiber scaffolds have been widely investigated for applications in neural tissue engineering due to their structural similarity to native extracellular matrices [1, 2]. Previous studies have shown that nanofiber topography can guide neural cell behavior, including cell adhesion, alignment, and neurite extension [3–6]. For example, aligned nanofibers have been reported to direct neural stem cell adhesion and neurite elongation, while surface functionalization with arginylglycylaspartic acid (RGD)-containing peptides enhances cell adhesion and proliferation [3]. Owing to their fibrous architecture and tunable properties, nanofiber scaffolds provide a platform for studying cell–material interactions relevant to neural systems.

Soy protein has recently been recognized as an attractive biomaterial for scaffold fabrication due to its biocompatibility and potential to influence cellular and immune-associated processes [7, 8]. Soy isoflavones and their metabolites have been reported to be associated with modulation of immune-related pathways in vivo [9, 10]. In our previous work, we developed collagen-based conduits incorporating soy protein isolate (SPI) and characterized their structural and biological properties [11]. The collagen-SPI conduits reduced the proliferation of human adult and fetal astrocytes and HMC3 cells, compared with collagen conduits in an in vitro analysis. We also found that collagen-SPI scaffolds reduced the gene expression of major histocompatibility complex class II (MHCII) molecules [12]. Investigating neural cell responses to SPI-containing nanofibers helps elucidate how these materials influence cellular behaviors and gene expression profiles relevant to neural environments.

Astrocytes play a central role in maintaining neural tissue homeostasis and responding to injury-related cues. They can contribute to tissue remodeling, secretion of trophic factors, and regulation of the extracellular environment, while also participating in processes such as glial scar formation [13, 14]. Following neural injury, fetal astrocytes have been reported to exhibit properties distinct from adult astrocytes, including the potential to create a more permissive cellular environment [15–17]. Additionally, fetal astrocytes may produce factors for neuron protection and therefore support neuron survival. Nanofibers made with SPI and collagen may influence cellular responses and gene expression patterns associated with immune-related processes. Given their dynamic migratory behavior observed in vitro, human fetal astrocytes provide a suitable model for investigating cell–biomaterial interactions.

In this study, we fabricated electrospun nanofibers composed of SPI, collagen, and polycaprolactone (PCL), a biocompatible polymer that enhances mechanical stability. Human fetal astrocytes were used as a model system to investigate cell–nanofiber interactions. We evaluated cellular responses including viability, proliferation, and migration, and performed RNA sequencing to characterize transcriptional changes associated with astrocyte interactions with SPI-containing nanofibers.

## Materials and Methods

### Nanofiber Fabrication

In this study, PCL (molecular weight of 80,000, Sigma-Aldrich, St. Louis, MO), SPI (MP Biomedicals, LLC, Santa Ana, CA), and type I bovine collagen were used to fabricate PCL, SPI/PCL, collagen(CO)/PCL and CO/SPI/PCL nanofibers by electrospinning. To generate the PCL fibers, PCL was dissolved in 1,1,1,3,3,3-Hexafluoro-2-propanol (Oakwood Products, Inc., Estill, SC) in a concentration of 6% (w/v). To generate the composite fibers, SPI (2%)/PCL (4%), collagen (3%)/PCL (4%), collagen (1%)/SPI (1%)/PCL (4%) were dissolved in 1,1,1,3,3,3-Hexafluoro-2-propanol to form solutions for electrospinning. Fibers were collected on aluminum foil that covered a mandrel placed 15 cm away from the needle tip of an infusion syringe with a flow rate of 0.48 ml/hr. The applied voltage for electrospinning was 15 kV. To generate the random fibers, the mandrel rotation speed was set at 100 RPM, while the rotation speed was increased to 1500 RPM to fabricate the aligned fibers. The standardized electrospinning parameters for each type of nanofibers were applied to generatenanofibers.

### Fiber Characterization by FTIR Assay, and Contact Angle Assay

Fourier transform infrared spectroscopy was performed to study the nanofiber component. Spectra were obtained using a PerkinElmer Spectrum 3 FT-IR Spectrometer (PerkinElmer, Waltham, MA), which is equipped with a crystal plate that combines a diamond and zinc selenide (ZnSe) element designed for use in attenuated total reflectance (ATR) spectroscopy. To obtain an infrared (IR) spectrum, the specimen was first loaded onto the ATR sample platform. A swivel press was used to ensure the sample had optimal contact with the ATR crystal. IR light was then directed to the crystal where it interacted with the sample. During this process, some of the IR is absorbed by the sample, and the remaining light is reflected back to the detector, thus creating an IR spectrum.

The contact angles of deionized water drops on nanofiber membranes were measured by a goniometer (ramé-hart instrument co., Succasunna, NJ) at room temperature, and water drop pictures were taken. The procedure was repeated on three samples of each type of nanofiber, and the mean contact angle was calculated.

### Fetal Astrocyte Growth on Nanofibers

The fetal human astrocytes (Catalog no.1800, ScienCell Research Laboratories, Carlsbad, CA) were cultured with fetal astrocyte medium (ScienCell Research Laboratories, Carlsbad, CA) in an incubator (37 °C) with 5% CO_2_. The nanofiber membrane on aluminum foil was cut as a round sample that fitted the 24-well plate or six-well plate wells.

### LIVE/DEAD® Cell Viability Assay

After the cells were cultured on randomly oriented nanofibers for two days, the cell viability was tested by LIVE/DEAD® cell vitality kit (Life Technologies Corp., Grand Island, NY). The staining cell solution was made by adding EthD-1 stock solution (2 µl of 2 mM) and calcein AM stock solution (2 µl of 1 mM) into a cell culture medium (1 ml). The cells were incubated with the staining solution for 25 min at 37 °C incubation before images were taken.

### SEM Assay for Nanofibers and Cell-Nanofiber Interaction

After the fetal astrocytes (10,000 cells/nanofiber sample) were grown on the nanofiber for two days, they were washed with phosphate buffered saline (PBS) and fixed with 4% paraformaldehyde. The graded ethanol was used to dehydrate the fixed samples and further dried with hexamethyldisilazane. The dried samples were coated with gold for SEM imaging. A scanning electron microscope (JSM-6460LV, JEOL Inc., Peabody, MA) was utilized to take images of the cells and the nanofibers. The fiber diameter of SEM images was analyzed by National Institutes of Health (NIH) ImageJ software (Bethesda, MD). The nanofibers without cell seeding were coated with gold and studied by SEM. The fiber diameter for each type of nanofibers was analyzed by NIH ImageJ software.

### Immunocytochemistry

The cells grown on different types of randomly oriented fibers were fixed with 4% paraformaldehyde. The cells were permeabilized with 0.5% Triton X-100 for 15 min and blocked with 10% FBS for 30 min. All the cells were dual-labeled with anti-GFAP (glial fibrillary acidic protein) and anti-S100B antibodies. The cells were incubated with monoclonal anti-GFAP antibody (BioLegend, San Diego, CA) and secondary Alexa Fluor® 594 donkey anti-mouse IgG (Jackson ImmunoResearch Laboratories, Inc., West Grove, PA) antibody. The cells were then incubated with polyclonal anti-S100B antibody (BioLegend, San Diego, CA) and secondary Alexa Fluor® 488 goat anti-rabbit IgG (Jackson ImmunoResearch Laboratories, Inc., West Grove, PA) antibody. Fluorescent images were taken using a fluorescent microscope (Zeiss Axio Observer).

Fluorescence images were acquired under identical acquisition settings for each channel, including illumination intensity, camera gain, and exposure time, using 12-bit image acquisition. Histograms and range indicators were examined to confirm that no pixels were saturated and that all measurements were within the linear detection range. Four independent replicates were performed for each fiber type.

The fluorescence intensity of both red (GFAP) and green (S100B) signals in labeled cells was measured and quantified using NIH ImageJ (Fiji). To account for background signal, scaffold-only controls were imaged under identical conditions for each fiber type and fluorescence channel. The background intensity per channel was measured and subtracted from all cell-associated fluorescence measurements. Secondary-antibody–only controls were also performed to assess nonspecific signal contribution and exhibited low fluorescence intensity relative to scaffold autofluorescence. For image analysis, regions of interest (ROIs) corresponding to individual cells were defined using the ROI Manager in ImageJ, and identical ROIs were applied across fluorescence channels for both individual intensity measurements and ratiometric analysis. The ratio of S100B (green) to GFAP (red) fluorescence within the same cell was calculated to reduce the influence of shared background noise and improve relative comparison between groups.

Due to the inherent autofluorescence of the nanofiber scaffolds and variability in fluorescence intensity, the resulting measurements are considered semi-quantitative, enabling relative comparisons between groups rather than absolute quantification of protein expression.

### Cell Cycle Study

The fetal astrocytes (150,000/nanofiber sample) were seeded on randomly oriented nanofiber samples fitting the six-well plate wells. After culturing for three days, the cells were collected by trypsin treatment and centrifugation. The cells were then fixed with cold (4 °C) ethanol (70%) for at least one hour. The samples were centrifuged, and the supernatant was decanted. The cells were washed with PBS and centrifuged to remove supernatant. The FxCycle™ propidium iodide ribonuclease (PI/RNase) staining solution (0.5 ml) was used to stain the cells of each sample for 30 min at room temperature. The cells were analyzed by a CytoFLEX flow cytometer (Beckman Coulter, Brea, CA). The flowcytometry data analysis was performed by FlowJo™ Software (FlowJo LLC, Ashland, OR).

### Cell Migration Analysis

The fetal astrocytes were grown on the nanofiber samples (10,000 cells/sample) for two days. The cells were then labeled with Invitrogen™ wheat germ agglutinin (WGA) with Alexa Fluor® 488 conjugation (5 µg/ml, Fisher Scientific International, Inc., Pittsburgh, PA) in cell culture medium for about one hour. The cell migration on nanofibers was recorded by a fluorescent microscope (Zeiss Axio Observer) covered by a plastic incubator with a temperature control (37 °C) and CO_2_ supply. Sequential fluorescent images were captured every five minutes for a continuous three hours. All studies were repeated at least three times. The cell migration was tracked by NIH ImageJ software and analyzed by Chemotaxis and Migration Tool programs for cell migration velocity and distance.

### Next Generation RNA Gene Sequencing Assay

Fetal astrocytes (150,000/sample) were cultured on randomly oriented CO/PCL or CO/SPI/PCL nanofibers that fit in the six-well plate wells. After culturing for three days, the RNA was extracted from the cultured cells with a Direct-zol™ RNA Microprep Kit (Zymo Research, Irvine, CA) for RNA sequencing assay. RNA extraction was performed from the cells grown on three samples of each type nanofibers.

The extracted RNA was analyzed by mRNA-Seq using the Illumina NovaSeq 6000 Sequencing System at the University of Kansas Medical Center Genome Sequencing Facility (Kansas City, KS). Total RNA (500 ng) was used to initiate the Universal Plus mRNA-seq with NuQuant stranded mRNA library preparation protocol (Tecan Genomics 0520-A01). A S1000 thermal cycler (Bio Rad) was used to process the total RNA fraction by oligo dT beads capture enrichment of mRNA, fragmentation of mRNA, reverse transcription into cDNA, end repair of cDNA, and ligation with the appropriate unique dual index (UDI) adaptors with strand selection and library amplification by polymerase chain reaction (PCR) (12 cycles). Library validation was performed using the D1000 ScreenTape Assay kit (Agilent Technologies 5067–5582) on the Agilent TapeStation 4200.

### Data Analysis of the RNA Gene Sequencing Assay

Sequencing reads were processed using the nfcore/rnaseq pipeline (v3.16.0 (doi: 10.5281/zenodo.1400710) [18] for quality control, alignment with STAR [19] and quantification with RSEM [20]. The merged gene counts were evaluated for differential expression using the rsem-run-ebseq function of standalone RSEM (v1.3.3). EBSeq uses a single Bayesian model to test all genes simultaneously, producing posterior probabilities of differential expression and eliminating the need for subsequent multiple testing correction [21]. The rsem-control-FDR function was used to select differentially expressed genes (DEGs), with false discovery rate (FDR) being defined as (1 – posterior probability of differential expression). All genes with FDR below 0.05 were defined as DEGs, regardless of fold change. Visualizations of RNASeq data were produced in R (R Core Team, 2023, R: A Language and Environment for Statistical Computing. R Foundation for Statistical Computing, Vienna, Austria). Normalized counts from EBSeq were used to calculate principal components with the prcomp function and the principal components analysis (PCA) plot was created with ggplot2 (Wickham 2016, ggplot2: Elegant Graphics for Data Analysis. Springer-Verlag, New York). A volcano plot based on differential expression results was produced with the plot function. The Database for Annotation, Visualization, and Integrated Discovery (DAVID) was used to identify KEGG signaling pathways enriched among up- and downregulated DEGs. Pathways with an adjusted p value less than 0.05 after multiple testing correction by the Benjamini-Hochberg method were considered significantly enriched. Heatmaps of DEGs associated with enriched pathways were created with the R pheatmap package (Kolde R. pheatmap: Pretty Heatmaps, R package version 1.0.12. 2020) using log2-transformed, normalized gene counts with Z-score scaling by gene. Samples and genes were hierarchically clustered based on Euclidean distance.

### Statistical Analysis

The data of research outcomes are described by mean ± standard deviation. The data was statistically analyzed by a two-tailed Student’s t-test and ANOVA using an SPSS program. A *p*-value of 0.05 is considered to be statistically significant.

## Results

### Characterization of Electrospun Nanofibers

**Fig. 1:**
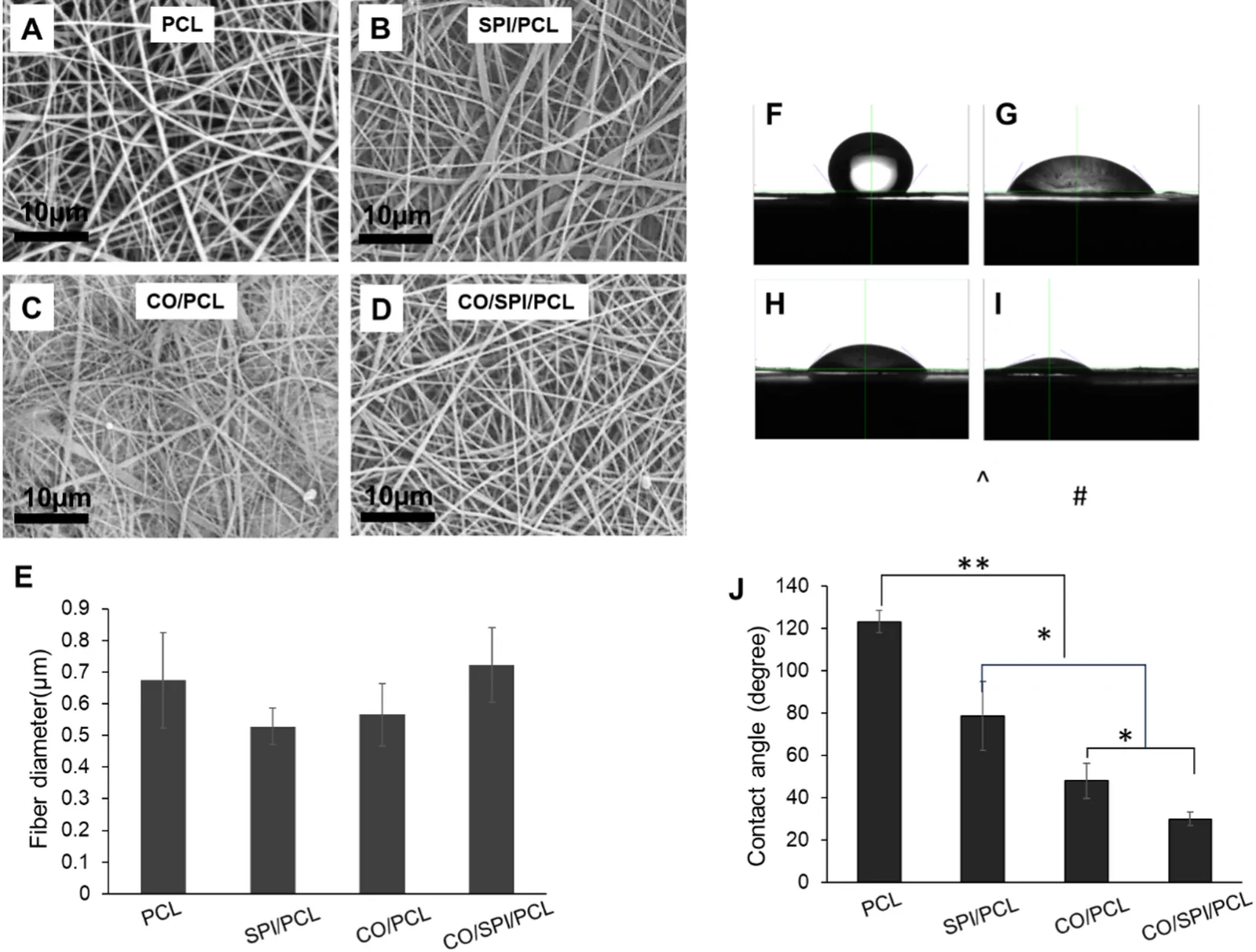
Fabricated nanofibers and measurement of contact angle for randomly oriented nanofibers. **A**–**D** SEM images of nanofibers: PCL (**A**), SPI/PCL (**B**), CO/PCL (**C**), and CO/SPI/PCL (**D**). **E** Quantification of nanofiber diameters. **F**–**I** Images showing contact angles of nanofibers: PCL (**F**), SPI/PCL (**G**), CO/PCL (**H**), and CO/SPI/PCL (**I**). **J** Quantification of nanofiber contact angle. ^*^, *p* < 0.01; ^**^, *p* < 0.05

Four types of nanofibers (PCL, SPI/PCL, collagen (CO)/PCL, and CO/SPI/PCL) were generated by electrospinning (Figs. 1A–D). The average fiber diameters of these four types of randomly oriented nanofibers are between 500 nm and 750 nm (Fig. 1E). The contact angle of PCL fibers (132° ± 5.2°) is significantly higher than the CO/PCL (78.7° ± 16.2°), SPI/PCL (48.0° ± 8.3°) and CO/SPI/PCL fibers (29.9° ± 3.2°) (< 0.05) (Figs. 1F–J). The collagen and SPI in the nanofibers reduced the fiber contact angle significantly.

**Fig. 2:**
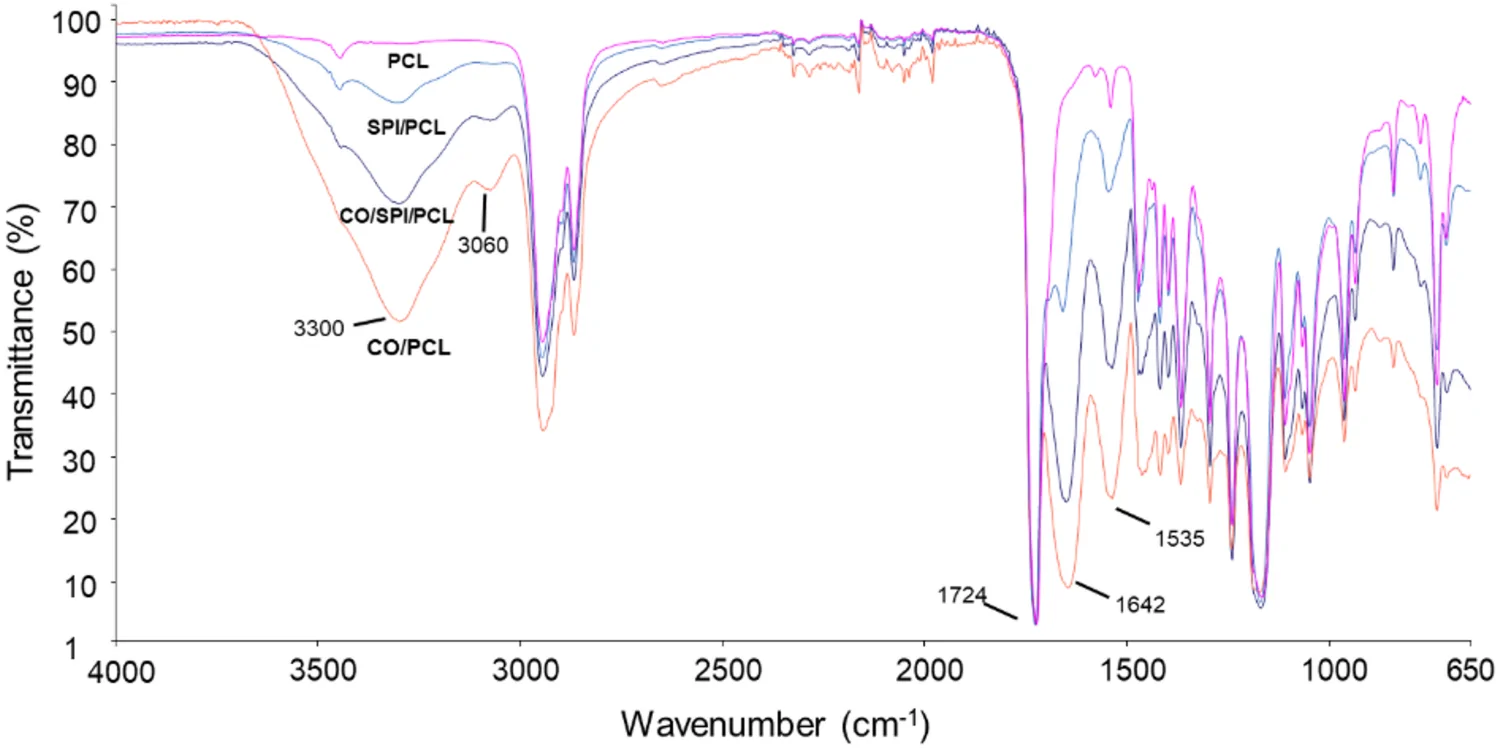
Normalized FTIR spectroscopy of randomly oriented nanofibers: PCL, SPI/PCL, CO/PCL, and CO/SPI/PCL nanofibers

Fourier transform infrared (FTIR) spectroscopy was used to determine the functional group of type I collagen, SPI, and PCL in the randomly oriented nanofibers (Fig. 2). This test shows the spectra obtained from FTIR for the different types of composite nanofibers. The intensified peaks at 1724 cm^− 1^ indicates the presence of PCL in the nanofibers. Both collagen and SPI components in the nanofibers showed peaks at 1530–1550 cm^− 1^ (N–H bending, amide II), and 1640–1660 cm^− 1^ (C = O stretching, amide I). The collagen showed peaks at 3300 cm^− 1^ (N–H bending, amide A), and 3060 cm^− 1^ (C = O stretching, amide B) [22–24].

### Fetal Astrocyte Viability, and Proliferation on Nanofibers

**Fig. 3:**
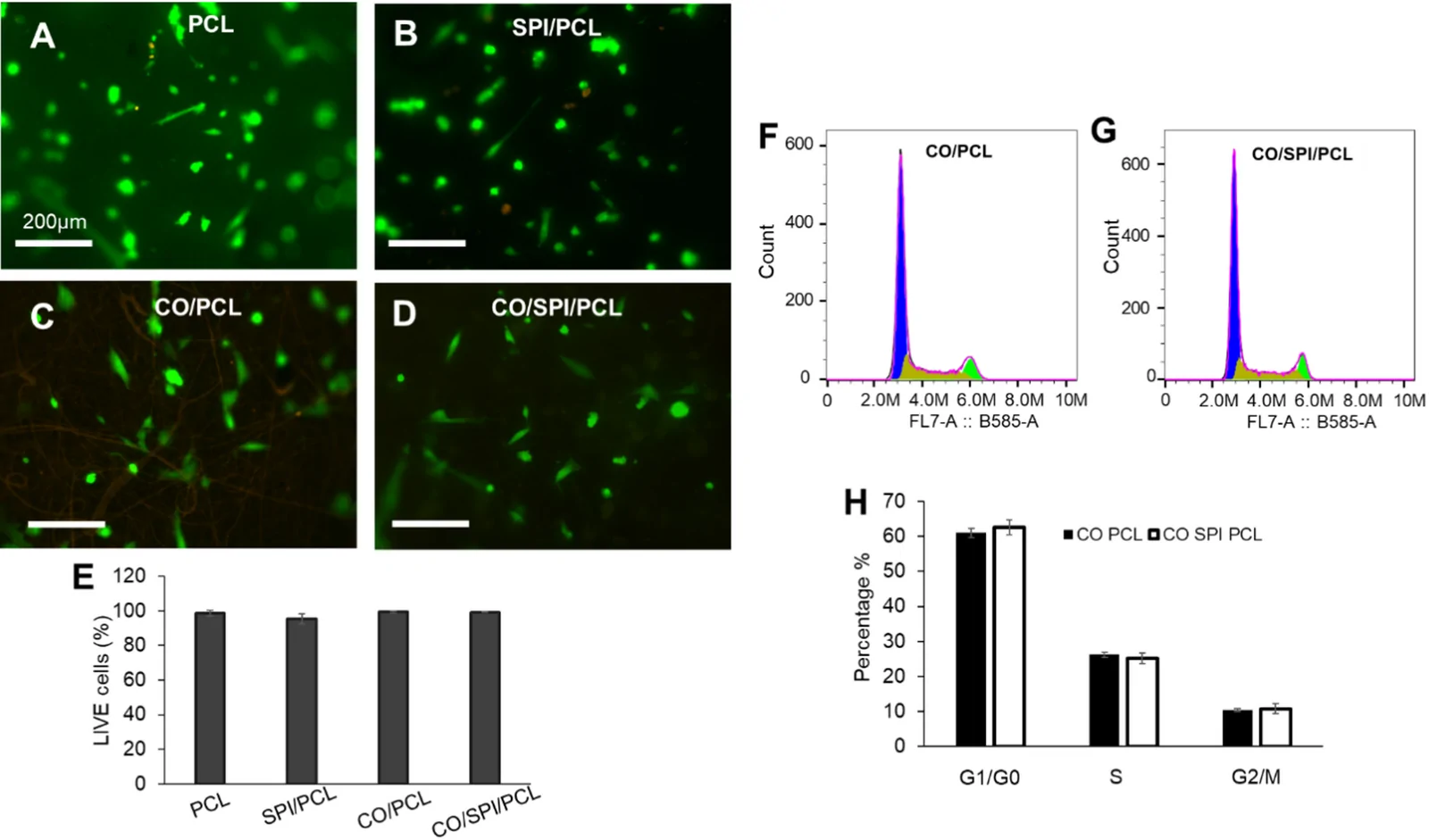
Cell viability and cell cycle assays for fetal astrocytes on randomly oriented nanofibers. **A**–**D** LIVE/DEAD® assay shows live cells (green) and dead cells (red) on nanofibers: PCL (**A**), SPI/PCL (**B**), CO/PCL (**C**), and CO/SPI/PCL (**D**). Scale bar, 200 μm. (**E**) Quantification of ratio of live cells to total cells. **F**–**H** Flow cytometry assay of fetal astrocytes on nanofibers: DNA content histogram of astrocytes on CO/PCL (**F**), DNA content histogram of astrocytes on CO/SPI/PCL (**G**), and histogram of cell cycle distribution of astrocytes at G1/G0, S, and G2/M phases (**H**)

**Fig. 4:**
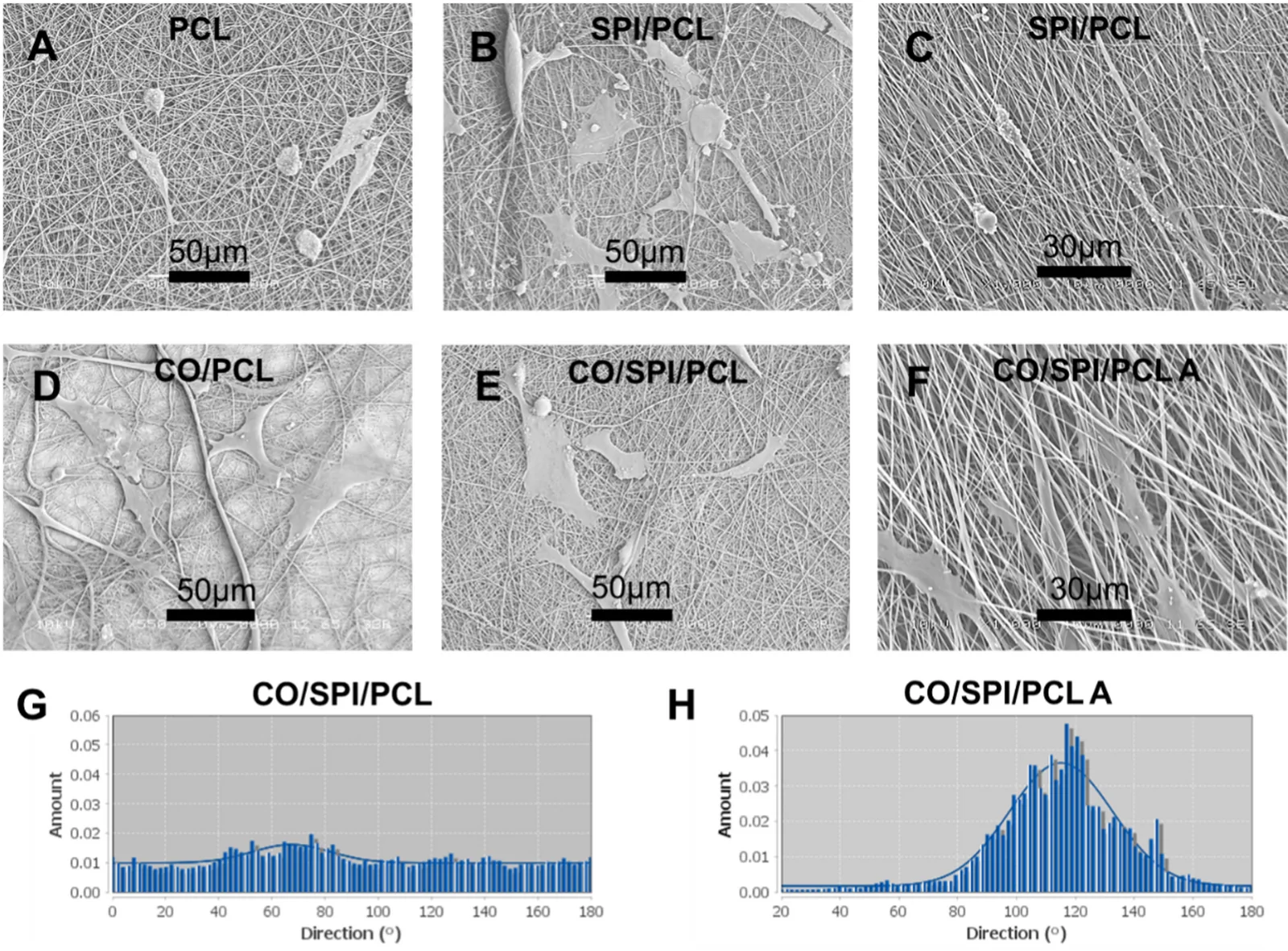
SEM images of fetal astrocytes on nanofibers matrices: **A** PCL random fiber, **B** SPI/PCL random fibers, **C** SPI/PCL aligned fibers, **D** CO/PCL random fibers, **E** CO/SPI/PCL random fibers, **F** CO/SPI/PCL aligned fibers. **G** The fiber directionality assay for fibers in figure E. **H** The fiber directionality assay for fibers in figure F. CO/SPI/PCL A, aligned CO/SPI/PCL nanofibers

In this study, we investigated the fetal astrocyte viability on nanofibers using a LIVE/DEAD® assay kit. Most cells grown on the randomly oriented nanofibers showed green fluorescence, indicating the live cells (Figs. 3A–D). Quantification of the cells showed high cell viability on all types of nanofibers (Fig. 3E). Flow cytometry was performed to study the cell cycle and proliferation on CO/PCL and CO/SPI/PCL fibers (Figs. 3F, G). The cell at G1 phase showed a similar percentage of cell number for astrocytes grown on CO/PCL (61.0% ± 1.3%) and CO/SPI/PCL fibers (62.5% ± 2.1%) (Fig. 3H). Scanning electron microscopy (SEM) images showed cell morphology on the nanofibers. The cells spread out and adhere to the random fibers (Figs. 4A, B, D, E). The cells extend out protrusions to attach to the nanofibers. The cells on aligned fibers showed elongated cell morphology and orientation along the fibers (Fig. 4C, F). The orientation assays for fibers in Fig. 4E and F were performed by the directionality function in ImageJ (Fiji) software (Fig. 4G, H). The aligned fibers show a sharp peak in histogram graph.

### The Analysis of S100B and GFAP in Fetal Astrocyte by Immunocytochemistry

**Fig. 5:**
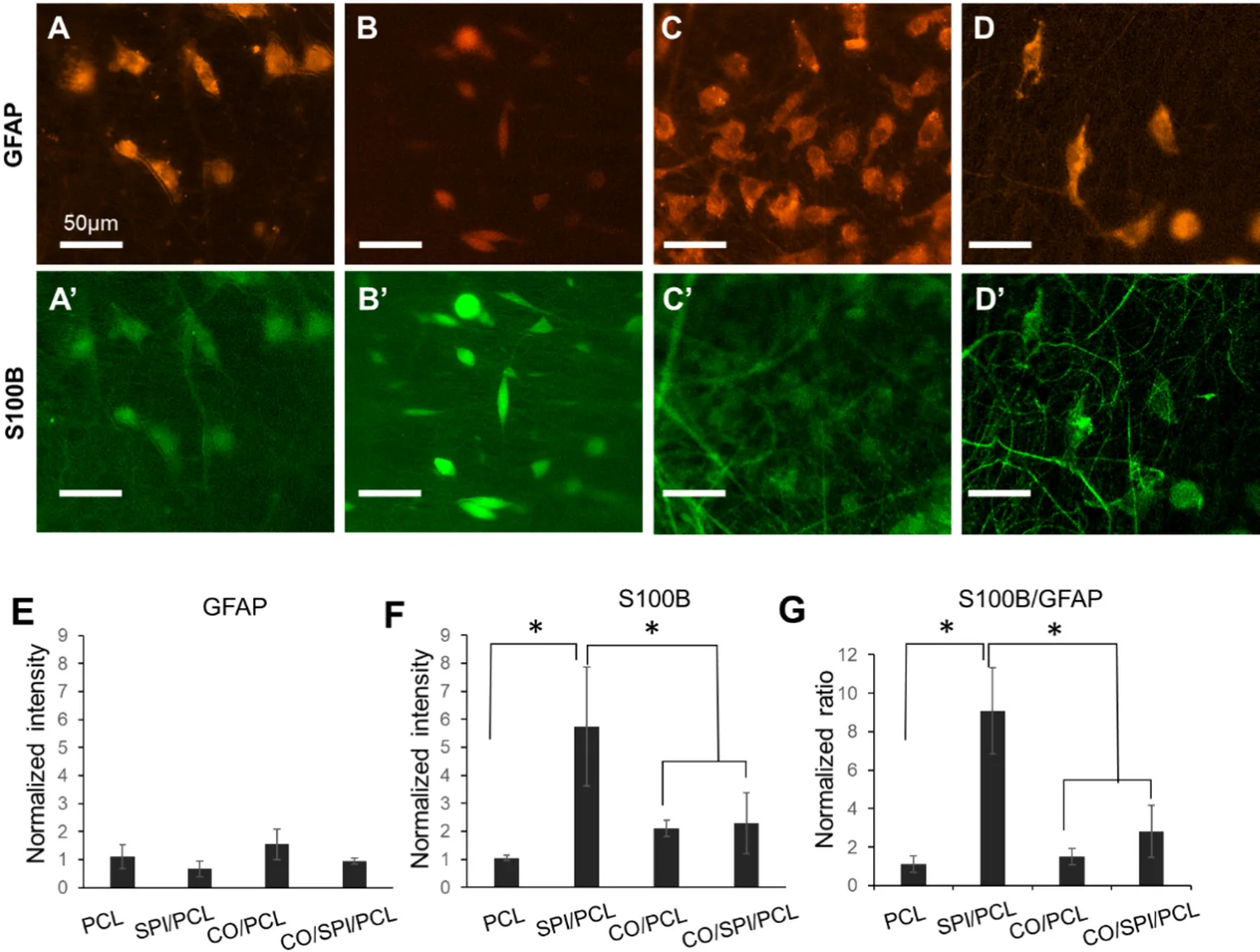
Immunostaining of S100B and GFAP for astrocytes grown on randomly oriented nanofibers. The cells were labeled with GFAP (**A**–**D**) and S100B (A’ to D’) antibodies on PCL (*n* = 199) (A, A’), SPI/PCL (*n* = 219) (B, B’), CO/PCL (*n* = 138) and CO/SPI/PCL (*n* = 162) nanofibers. The normalized fluorescent intensities show the expression of GFAP (**C**) and S100B (**D**) in astrocytes on nanofibers. **E** The normalized ratio of red to green fluorescent intensity shows the relative ratio of GFAP (red) versus S100B (green) in astrocytes on nanofibers. *, *p* < 0.05; n, cell number

In this study, immunocytochemistry was performed for dual labeling of GFAP and S100B for fetal astrocytes on randomly oriented fibers (Fig. 5A–D). S100B and GFAP are specific markers of astrocyte. The study aims to study the expression of these markers on different types of nanofibers. Four independent samples were analyzed in this study. The fluorescent intensity of the two markers in astrocytes was measured and quantified by ImageJ. One-way ANOVA was used to compare the means of quantified fluorescent data of different experimental groups. The normalized S100B level in astrocytes on CO/SPI/PCL fibers was significantly higher than the cells on other types of fibers (*p* < 0.05, Fig. 5F), while the normalized GFAP level was not significantly different for cells on different types of fibers (Fig. 5E). We further quantified the ratio of S100B to GFAP in cells on nanofibers. The S100B to GFAP ratio in cells on SPI/PCL was significantly higher than all the other groups (*p* < 0.01, Fig. 5G).

### Characterization of Fetal Astrocyte Migration on Nanofibers

**Fig. 6:**
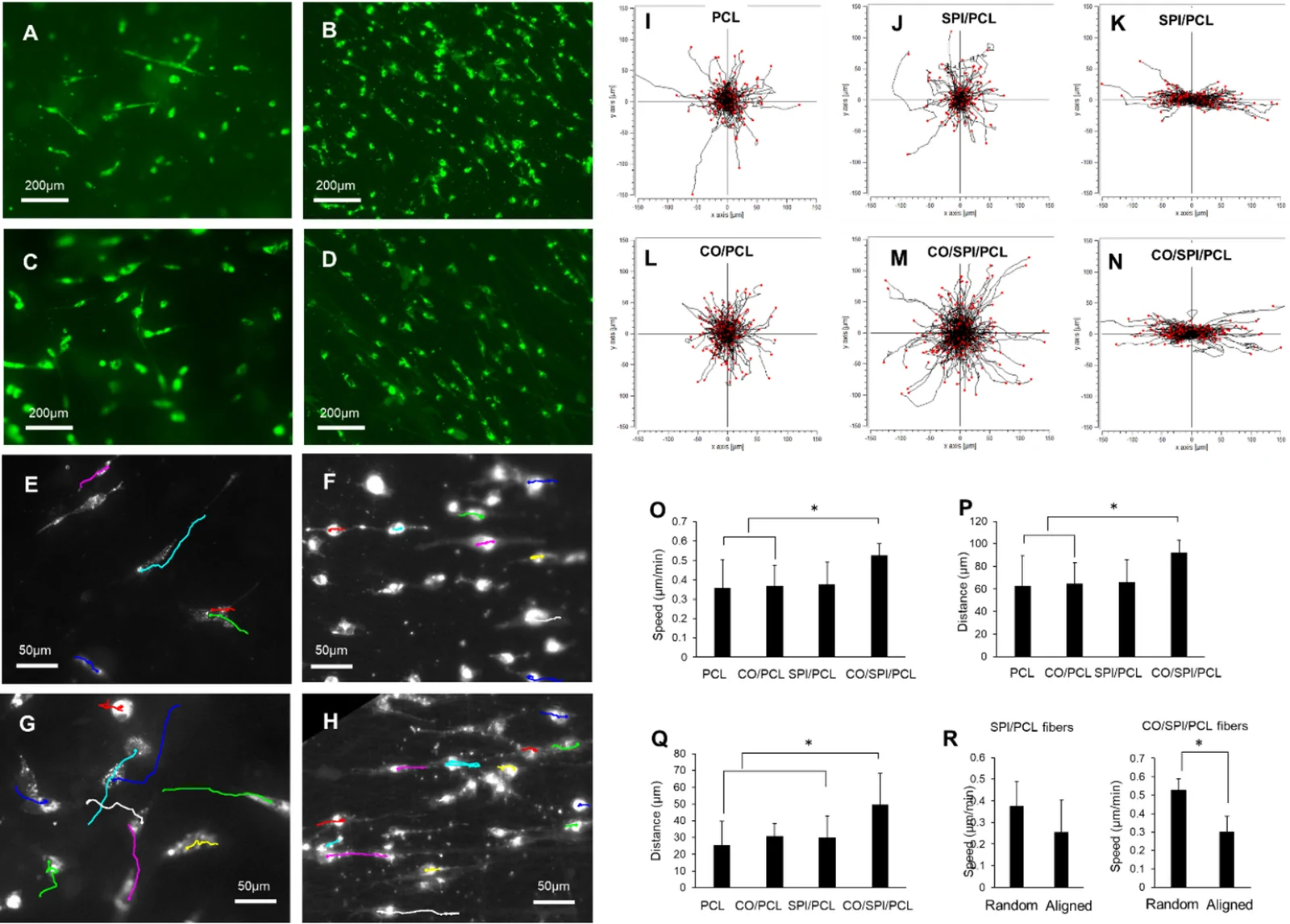
Analysis of astrocyte migration on nanofibers. **A**, **C** Growth of astrocytes on random nanofibers generated by SPI/PCL (**A**) and collagen/SPI/PCL (**C**). **B**, **D** Growth of astrocytes on aligned nanofibers generated by SPI/PCL (**B**) and collagen/SPI/PCL (**D**). **E**, **G** Tracking of astrocyte migration on aligned nanofibers generated by SPI/PCL (**E**) and collagen/SPI/PCL (**G**). **F**, **H** Tracking of astrocyte migration on aligned nanofibers generated by SPI/PCL (**F**) and CO/SPI/PCL (**H**). Cells labeled with WGA with Alexa Fluor® 488 conjugation. Scale bar, 200 μm. **I**–**N** Tracks of cell migration from eight experiments, each line indicates one cell migration pathway, initial positions of all cells are in the center of each frame: Cell migration tracks on random fibers (I, *n* = 195; J, *n* = 151; L, *n* = 213; M, *n* = 254), and cell migration tracks on aligned CO/SPI/PCL fibers (**K**, *n* = 208; N, *n* = 234). n, cell number. **O** Quantification of cell migration speed. **P** Quantification of accumulated cell migration distance. **Q** Quantification of Euclidean cell migration distance. **R** Comparison of cell migration speeds on random and aligned fibers. *, < 0.05

The cell growth and morphology on nanofibers were studied after the cells were labeled with Alexa Fluor™ 488 conjugated wheat germ agglutinin (WGA). Eight replications were performed based on eight independently prepared samples. One time-lapse recording of cell migration was taken for each sample in this study. These cells showed random orientation on random SPI/PCL (Fig. 6A) and CO/SPI/PCL nanofibers (Fig. 6C), while the cells showed oriented morphology on aligned SPI/PCL (Fig. 6B) and CO/SPI/PCL nanofibers (Fig. 6D). The cells migrated dynamically on both random nanofibers (SPI/PCL fibers, Fig. 5E, see also supplemental material video 1; and CO/SPI/PCL fibers, Fig. 6G, see also supplemental material video 2) and aligned nanofibers (SPI/PCL, Fig. 6F, see also supplemental material video 3; and CO/SPI/PCL, Fig. 6H, see also supplemental material video 4). The cell migration tracks are labeled with colored lines (Figs. 6I–N). One-way ANOVA was used to compare the means of quantified cell migration data of different random nanofiber groups. The cell migration speed on CO/SPI/PCL nanofibers (0.53 ± 0.06 μm/min) is significantly higher than that on CO/PCL (0.37 ± 0.11 μm/min) and PCL (0.36 ± 0.15 μm/min) nanofibers (Fig. 6O). The accumulated and Euclidean distance of cell migration were quantified (Fig. 6P). The Euclidean distance of cell migration on PCL and SPI/PCL nanofibers is significantly lower than that on CO/SPI/PCL fibers (Fig. 6Q). The cells showed a significantly higher migration speed on CO/SPI/PCL random fibers compared with aligned fibers (Fig. 6R). The migration speeds on aligned SPI/PCL and CO/SPI/PCL fibers are 0.25 ± 0.15 μm/min and 0.3 ± 0.08 μm/min respectively, while the migration speeds on random SPI/PCL and CO/SPI/PCL fibers are 0.38 ± 0.11 μm/min and 0.53 ± 0.06 μm/min, respectively.

### Differential Gene Expression of Astrocytes on Nanofibers

**Fig. 7:**
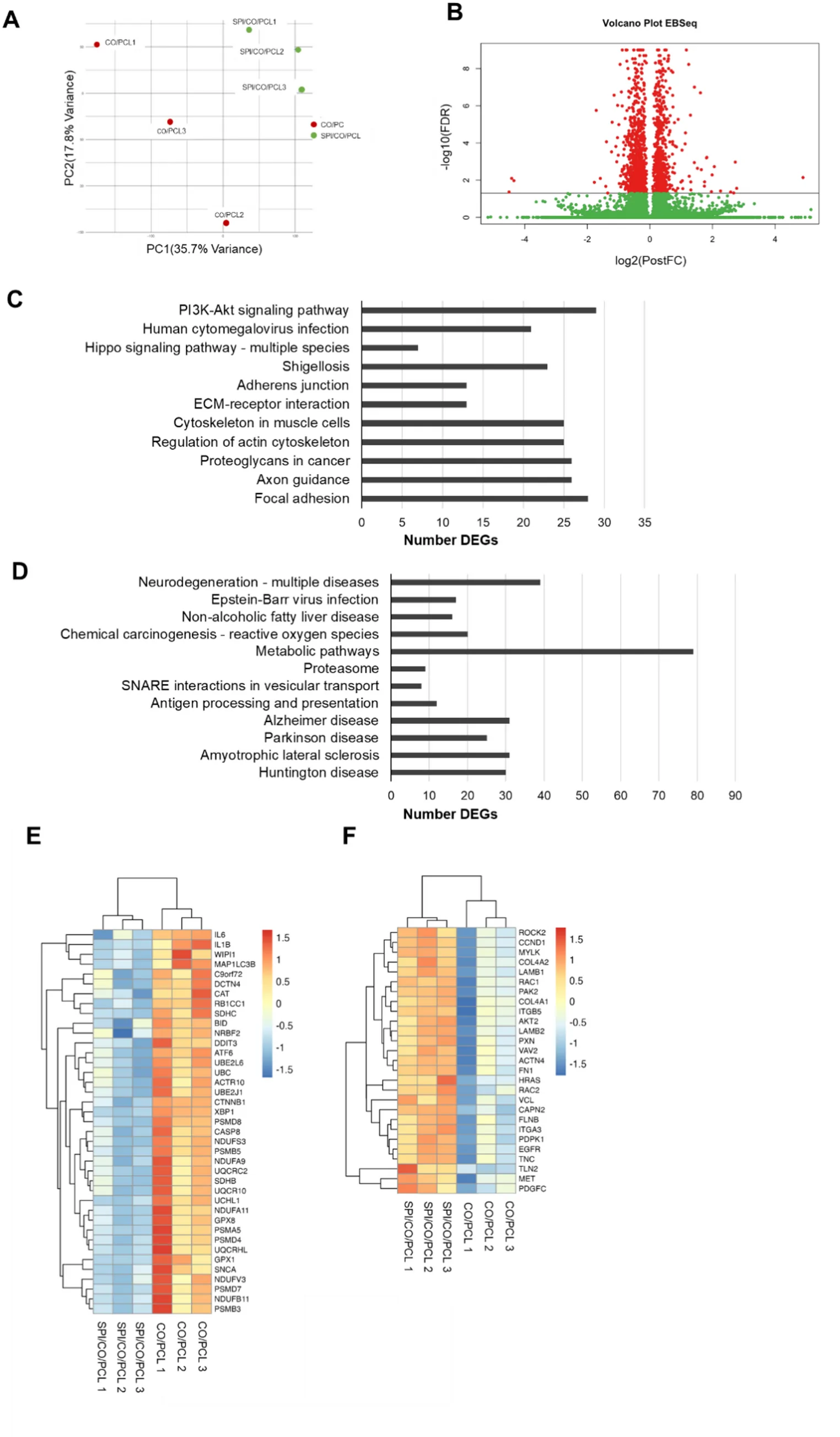
Analysis of RNA sequencing data. **A** Two-dimensional Principal Components Analysis (PCA) plot of RNA-seq samples based on normalized gene counts. Samples are color-coded by nanofiber type. **B** Volcano plot with log2(Posterior Fold Change) on the X axis and -log10(False Discovery Rate (FDR)) on the Y axis. Each point represents a gene, with red indicating genes that are significantly differentially expressed (FDR < 0.05) in fetal astrocytes on CO/SPI/PCL versus CO/PCL nanofibers and green denoting those that are not. Points to the right of the midline are up-regulated in cells on CO/SPI/PCL cells relative to cells on CO/PCL, while those to the left are down-regulated in cells on CO/SPI/PCL. **C** KEGG pathways enriched among up-regulated DEGs in astrocytes on CO/SPI/PCL nanofibers relative to cells on CO/PCL fibers. **D** KEGG pathways enriched among down-regulated DEGs in astrocytes on CO/SPI/PCL nanofibers relative to cells on CO/PCL fibers. Heatmaps of DEGs in the KEGG Neurodegeneration – multiple diseases (**E**) and Focal adhesion (**F**) pathways. The color scale represents the Z-score calculated across each gene. Genes (rows) and samples (columns) are clustered by Euclidean distance

To compare gene expression in astrocytes grown on randomly oriented CO/SPI/PCL nanofibers versus those grown on CO/PCL nanofibers, we performed RNA-Seq on three samples from each condition. The total number of clean reads per RNA-Seq library ranged from 59.3 million to 66.5 million for cells on CO/PCL and from 55.2 million to 70.2 million for cells on CO/SPI/PCL. Over 95% of the reads from all samples mapped to the human genome (GRCh38), with over 85% aligning uniquely. Principal components analysis (PCA) of normalized gene expression showed a clear separation of samples from cells on CO/PCL and CO/SPI/PCL nanofibers (Fig. 7A). Differential gene expression analysis identified 652 up-regulated and 835 down-regulated genes (FDR < 0.05) in astrocytes on CO/SPI/PCL nanofibers relative to those on CO/PCL nanofiber (Fig. 7B).

### Analysis of Differentially Expressed Genes

**Fig. 8:**
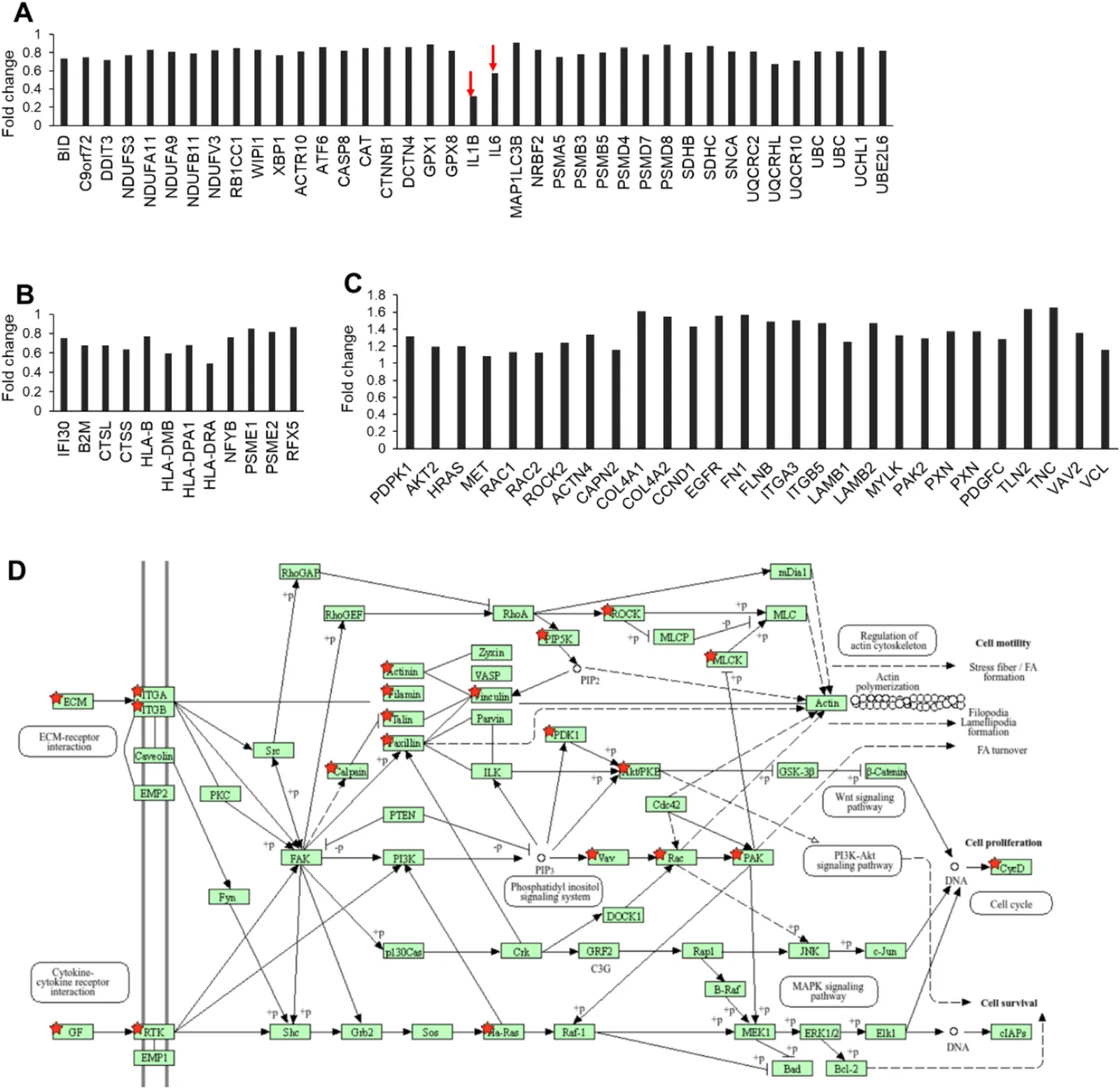
Fold changes of DEGs in enriched KEGG signaling pathways. DEGs in enriched KEGG signaling pathways of neurodegeneration (**A**), and antigen processing and presentation (**B**), which are significantly down-regulated in fetal astrocytes grown on collagen/SPI/PCL fibers compared with collagen/PCL. (**C**) DEGs in the enriched KEGG signaling pathway of focal adhesion, which are significantly up-regulated in fetal astrocytes grown on collagen/SPI/PCL fibers compared with collagen/PCL. (**D**) KEGG pathway of focal adhesion; red stars indicate up-regulated genes

Up- and down-regulated DEGs were separately analyzed for enrichment of Kyoto Encyclopedia of Genes and Genomes (KEGG) pathways. Our results showed that 11 signaling pathways are enriched among up-regulated genes (Fig. 7C), and 12 pathways are enriched among down-regulated genes (Fig. 7D). The pathways enriched among down-regulated genes include a few pathways that regulate neurodegeneration such as Parkinson’s disease, Alzheimer disease, amyotrophic lateral sclerosis, and Huntington’s disease. The down-regulated DEGs (39 DEGs) summarized in the “neurodegeneration - multiple diseases” pathway are involved in the processes of these diseases (Figs. 7E and 8A and supplemental material Table 1). In particular, the assay shows the significant down-regulation of IL1B and IL6 genes by the astrocytes on CO/SPI/PCL fibers compared with the cells on CO/PCL fibers. The down-regulated pathways also include “antigen processing and presentation” (12 DEGs) (Fig. 8B and supplemental material Table 2). The DEGs in this pathway include four human leukocyte antigen (HLA) genes (HLA-B, HLA-DMB, HLA-DPA1, and HLA-DRA). These genes encode molecules that regulate CD4^+^ T cell function and therefore play a critical role in the immune reaction and autoimmune diseases. The “focal adhesion” pathway, which is enriched among up-regulated genes (28 DEGs), includes several genes (COL4A1, COL4A2, FN1, LAMB1, LAMB2, AKT2, RAC1, RAC2, ROCK2, PIP5K1A) that encode molecules likely to regulate astrocyte adhesion and motility on CO/SPI/PCL substrate (Figs. 7F and 8C and D and supplemental material Table 3).

**Table 1:** Representative differentially expressed genes (DEGs) associated with enriched pathways in fetal astrocytes cultured on CO/SPI/PCL versus CO/PCL nanofibers, identified by EBSeq analysis

	Gene name	PPEE	Log2FC
Neurodegeneration Pathway	BH3 interacting domain death agonist(BID)	4.48E-06	−0.445362
	DNA damage inducible transcript 3(DDIT3)	4.88E-06	−0.4745164
	NADH: ubiquinone oxidoreductase core subunit S3(NDUFS3)	7.73E-11	−0.370124
	X-box binding protein 1(XBP1)	0	−0.3733702
	interleukin 1 beta(IL1B)	0.002734	−1.6417523
	interleukin 6(IL6)	3.42E-12	−0.8017297
	activating transcription factor 6(ATF6)	1.46E-08	−0.2183717
	caspase 8(CASP8)	2.02E-06	−0.2861564
	glutathione peroxidase 1(GPX1)	1.71E-07	−0.1646467
	microtubule associated protein 1 light chain 3 beta(MAP1LC3B)	0.000119	−0.1404295
	succinate dehydrogenase complex iron sulfur subunit B(SDHB)	0.012927	−0.3189196
	synuclein alpha(SNCA)	0.000649	−0.2994271
Antigen processing and presentation pathway	IFI30 lysosomal thiol reductase(IFI30)	1.61E-06	−0.406738
	beta-2-microglobulin(B2M)	0.03148	−0.5666004
	cathepsin S(CTSS)	3.39E-06	−0.6472505
	major histocompatibility complex, class I, B(HLA-B)	1.54E-05	−0.3737758
	major histocompatibility complex, class II, DP alpha 1(HLA-DPA1)	0	−0.5482376
	major histocompatibility complex, class II, DR alpha(HLA-DRA)	0	−1.0247279
	regulatory factor X5(RFX5)	0.001237	−0.2040137
Focal adhesion pathway	AKT serine/threonine kinase 2(AKT2)	0.039633	0.2594486
	Rac family small GTPase 1(RAC1)	0.003577	0.1770831
	Rho associated coiled-coil containing protein kinase 2(ROCK2)	2.21E-05	0.3083014
	collagen type IV alpha 1 chain(COL4A1)	0.003078	0.6905409
	epidermal growth factor receptor(EGFR)	5.86E-06	0.6389
	fibronectin 1(FN1)	0.002261	0.6454797
	integrin subunit alpha 3(ITGA3)	0.000546	0.5849219
	integrin subunit beta 5(ITGB5)	0.015042	0.5551915
	paxillin(PXN)	0.032551	0.4565996
	talin 2(TLN2)	7.78E-05	0.7100066
	vinculin(VCL)	0.000237	0.2084969

Genes are grouped by pathway, including neurodegeneration, antigen processing and presentation, and focal adhesion–related pathways. Log2 fold change (log2FC) values indicate the magnitude of expression changes. Statistical significance is reported as posterior probability of equivalent expression (PPEE), which represents the EBSeq-derived false discovery rate (FDR), calculated as (1 – posterior probability of differential expression)

Representative differentially expressed genes (DEGs) associated with key enriched pathways, including, neurodegeneration, antigen processing and presentation and focal adhesion, are summarized in Table 1. Genes are reported with corresponding log2 fold changes and adjusted p values (FDR) based on EBSeq analysis.

## Discussion

Biomaterial neural conduits have been reported in numerous studies for spinal cord and peripheral nerve repair [25–27]. The structural design of a neural bioscaffold is the research target to provide a platform for studying cell–material interactions relevant to neural systems. Nanofiber scaffolds have been widely studied as biomimetic platforms because they mimic the structure of extracellular matrix structure and axonal morphology [28–30]. Soy protein has shown promising application in tissue repair [7, 31, 32]. However, research into the interactions between neural cells and SPI-based nanofibers remains limited. In this study, we fabricated PCL, SPI/PCL, CO/PCL and CO/SPI/PCL nanofibers. The FTIR assay indicated the different fiber components. We also found that combining soy protein and collagen with PCL in the composite nanofiber can result in a lower contact angle compared to PCL blended with either protein alone. The study indicates that the hydrophilic, polar groups in both proteins may complement each other and the synergistic effect leads to increased wettability. In our previous study, we reported the fabrication of a SPI-collagen hybrid protein neural conduit. SPI biocompatibility and the effect of SPI on astrocyte proliferation have been reported in our previous study [33]. Fetal astrocytes showed a lower cell proliferation rate when they were grown in SPI/collagen hydrogel compared with collagen hydrogel, thereby indicating that the SPI component can reduce cell proliferation. This study investigated the influence of SPI-based fibers on the cellular activity and transcriptional profile of human fetal astrocytes. The observed outcomes are associated with the inclusion of SPI in the nanofiber composition, together with differences in physical properties such as surface wettability. Chemical composition and physical properties are inherently coupled in this material system and were not independently controlled. Therefore, the observed cellular responses are interpreted as arising from the combined influence of these factors.

In this study, we observed that the fetal astrocytes showed high cell viability on CO/SPI/PCL nanofibers and CO/PCL nanofibssers. We also found that the G1, S, and M phases in the cell cycle of fetal astrocytes on CO/PCL or CO/SPI/PCL were not significantly different, indicating that the SPI component in the nanofiber does not affect the cell cycle. Cyclin A regulates DNA replication at S-phase and mitosis. Cyclin B1 and B2 activate the cell transition from G2 phase to mitosis [34]. Cyclin D1 controls the cell transition from the G1 phase to the S phase in a cell cycle [35]. The RNA sequencing data showed that the folds for the gene expression of CCNB1, CCNB2, and CCNA2 are 1.25, 1.17, and 1.08 respectively for the cells on CO/PCL versus CO/SPI/PCL fibers. However, the fold for the gene expression of CCND1 is 0.68 for the cells on CO/PCL versus CO/SPI/PCL fibers. The combined function of these gene expressions may explain that the cells do not show significant difference at different phases in their cell cycles on CO/PCL and CO/SPI/PCL nanofibers.

Although both GFAP and S100B are astrocyte markers, they express at different stages of neural development [36]. GFAP is expressed at early stage in neural development, while S100B is expressed at more mature stage of astrocytic development state [36]. In this study, we observed that the fluorescent intensities of S100B and GFAP are not significantly different between CO/PCL and CO/SPI/PCL groups. The finding is consistent with the RNA sequencing data that showed that the gene expression of S100B and GFAP was not significantly different between CO/PCL and CO/SPI/PCL groups. It is interesting to observe a higher S100B/GFAP ratio in astrocytes grown on SPI/PCL nanofibers compared with other nanofibers. This shift may be associated with changes in astrocyte phenotype under different material conditions. GFAP is an intermediate filament protein involved in structural stability, whereas S100B is a calcium-binding protein associated with intracellular signaling, metabolism, and neurotrophic-related functions. Previous studies have reported that S100B is associated with neuroprotective processes, including reduced microgliosis and decreased tumor necrosis factor (TNF-α) production [37, 38]. Our results indicate that SPI/PCL nanofibers are associated with differences in S100B expression in fetal astrocytes at the transcriptional level, highlighting their potential in regulating fetal astrocyte maturation. Further research is essential to elucidate the specific role of soy protein in fostering fetal astrocyte function and its downstream impact on neural repair.

Cell migration is an important cellular activity in the tissue regeneration process [39–42]. Cell migration has been observed on nanofibers, and the topography of nanofiber matrices can significantly affect cell morphology and migration [43, 44]. In this study, we observed the effect of material component in nanofibers on fetal astrocyte migration. The fetal astrocyte motility is an important cellular behavior because it represents the capability of cell distribution within the lesion after they are grafted. In a previous study, we found that the fetal astrocytes migrated dynamically on SPI/collagen at a migration speed of 0.64 ± 0.08 μm/min, and collagen substrates at a migration speed of 0.61 ± 0.07 μm/min. The cell migration velocities were not significantly different on those matrices. In this study, we found that the cell migration velocity on CO/SPI/PCL fibers is significantly higher than that on CO/PCL fibers.

A number of studies have reported that the aligned nanofibers can guide the orientation of cell growth [1, 45, 46]. It was also reported that the axon migration was guided by the fibers [47]. As shown in these studies, we observed the fetal astrocyte orientation along the aligned fibers. The cell migration on nanofibers was reported in previous studies [48–50]. The glioma cells showed higher migration speed on aligned fibers than random PCL nanofibers [48]. In another study, SH-SY5Y cells showed higher cell coverage on random PCL fibers than aligned fibers after culturing for 7 days, suggesting that random fibers supported efficient surface coverage, migration, and intercellular communication [50]. In this study, we report additional observations of the interaction of fetal astrocytes with nanofibers and their impact on cell motility. We compared cell migration on random and aligned nanofibers and found the aligned nanofibers directed the cell orientation of adhesion and guided the cell migration. Interestingly, the migration velocities of fetal astrocytes on aligned CO/SPI/PCL nanofibers fibers were significantly lower than the cells on random fibers. The oriented migration may allow more effective cell translocation between the lesion and healthy neural tissue. This could indicate that the primary function of fiber alignment is to guide cell movement, but not necessarily to accelerate cell migration speed. Even if cells move slower, they move efficiently toward a target in neural regeneration process rather than moving randomly. These studies suggest that different cell types migrate on nanofibers in a specific manner, with their motility influenced by the structural and component properties of the scaffolds. In this study, the collagen and SPI in the nanofibers are not crosslinked. The degradation rate of collagen/SPI nanofibers can be tailored for implantation by modulating the crosslinking density with 1-ethyl-3-(3-dimethylaminopropyl)carbodiimide (EDC), often combined with N-hydroxysuccinimide (NHS) for higher efficiency. This biocompatible crosslinking approach stabilizes the scaffold structure, allowing for controlled biodegradation rates suitable for neural tissue engineering.

In this study, we observed that up-regulated genes in cells grown on CO/SPI/PCL nanofibers were enriched in focal adhesion pathway genes that regulate cell adhesion and migration. Inclusion of the SPI component is associated with higher gene expression of extracellular matrix (ECM) proteins including collagen type IV alpha 1 chain (COL4A1) and collagen type IV alpha 2 chain (COL4A2), fibronectin 1(FN1), laminin subunit beta 1(LAMB1), and laminin subunit beta 2(LAMB2). These ECM proteins are critical molecules that mediate cell adhesion and migration. Collagen can be recognized by cell integrin receptors and provide structural support for cell attachment. Fibronectin has a collagen-binding site and can organize the ECM components. Fetal astrocytes also increased the integrin subunit gene expression including integrin subunit alpha 3 (ITGA3), beta 5 (ITGB5), beta 1 (LAMB1), and beta 2 (LAMB2). The cell surface receptors are crucial in the cell-ECM interaction. The increased expression is consistent with the observed increase in cell migration. Both focal adhesion and the PI3K-AKT signaling pathway are enriched in up-regulated genes. The up-regulated genes in both pathways include AKT serine/threonine kinase 2(AKT2) [51], Rac family small GTPase 1(RAC1), Rac family small GTPase 2(RAC2) [52], Rho-associated coiled-coil-containing protein kinase 2(ROCK2) [53], and phosphatidylinositol-4-phosphate 5-kinase type 1 alpha (PIP5K1A). These molecules are involved in the regulation of cell morphology and migration. AKT and PIP5K are important for cell survival. Phosphatidylinositol 4-phosphate 5-kinase (PIP2) generated by PIP5K is involved in cell membrane dynamics and cell motility. AKT is a key molecule regulating cell growth and inhibition of cell apoptosis. Rock2, referred to as Rho-associated protein kinase, and Rac2, a small GTPase protein, are key molecules that regulate cytoskeleton organization, which is essential for cell motility, cell morphology, and cell adhesion. In this study, we observed the up-regulation of those genes, suggesting that the SPI component in nanofibers is associated with increased expression of genes related to cell adhesion, morphology, and migration, consistent with the observed cellular behavior. We also observed the higher migration distance for fetal astrocytes on CO/SPI/PCL nanofibers compared with CO/PCL fibers. The higher gene expression of the above molecules may explain the higher cell migration speed.

KEGG pathways that are enriched among down-regulated genes are “neurodegeneration-multiple diseases” and “antigen processing and presentation.” In particular, we observed the down-regulation of IL1B and IL6 genes. Both IL1β and IL6 were reported to function as pro-inflammatory cytokines that drive neuroinflammation and secondary neuronal death after SCI [54]. The down-regulation of major histocompatibility complex genes HLA-B, HLA-DMB, HLA-DPA1, and HLA-DRA will reduce the activation of CD4^+^ T cells [55, 56]. In a previous study, we studied the gene expression of human microglia on collagen or CO/SPI substrates by RNA-Seq. We reported that among down-regulated genes, the “antigen processing and presentation” pathway showed enrichment, primarily due to the down-regulation of MHCII molecules [12]. These in vitro findings establish a foundational basis for subsequent in vivo studies to validate the role of nanofibers containing SPI in mediating immune responses.

## Supplementary Information

Below is the link to the electronic supplementary material.

Supplementary material 1 (AVI 636.6 kb)—fetal astrocyte migration on random SPI/PCL fibers

Supplementary material 2 (AVI 1065.8 kb)—fetal astrocyte migration on random CO/SPI/PCL fibers

Supplementary material 3 (AVI 1256.2 kb)—fetal astrocyte migration on aligned SPI/PCL fibers

Supplementary material 4 (AVI 1220.4 kb)—fetal astrocyte migration on aligned CO/SPI/PCL fibers

Supplementary material 5 (DOCX 28.4 kb)—DEGs belonging to enriched KEGG signaling pathways

## Data Availability

All data generated or analysed during this study are included in this published article.
