# Supplementary material for: Investigating the Cellular Activity and Differential Gene Expression of Human Astrocytes in Interaction with Protein Composite Nanofibers
Source: Neurochem Res. 2026 Aug 14;51(4):238. doi: 10.1007/s11064-026-04855-y (PMC13476280; doi:10.1007/s11064-026-04855-y)
Supplement: Supplementary file 1 [file 11064_2026_4855_MOESM5_ESM.docx]

Table 1. Significantly downregulated DEGs (PPEE < 0.05) belonging to the enriched neurodegeneration pathway in fetal astrocytes cultured on CO/SPI/PCL versus CO/PCL nanofibers, analyzed via EBSeq. Log2FC values are included to indicate magnitude of downregulation. PPEE (Posterior Probability of Equivalent Expression) can be considered equivalent to FDR (False Discovery Rate), meaning that no additional multiple testing correction is required.

| ENSEMBL GENE ID | Gene Name | | | | PPEE | Log2FC |
| --- | --- | --- | --- | --- | --- | --- |
| ENSG00000015475 | BH3 interacting domain death agonist(BID) | |  |  | 4.48E-06 | -0.445362 |
| ENSG00000147894 | C9orf72-SMCR8 complex subunit(C9orf72) | |  |  | 0.012375 | -0.4194859 |
| ENSG00000175197 | DNA damage inducible transcript 3(DDIT3) | |  |  | 4.88E-06 | -0.4745164 |
| ENSG00000213619 | NADH:ubiquinone oxidoreductase core subunit S3(NDUFS3) | | | | 7.73E-11 | -0.370124 |
| ENSG00000174886 | NADH:ubiquinone oxidoreductase subunit A11(NDUFA11) | | |  | 6.54E-05 | -0.2623323 |
| ENSG00000139180 | NADH:ubiquinone oxidoreductase subunit A9(NDUFA9) | | |  | 0.015671 | -0.304717 |
| ENSG00000147123 | NADH:ubiquinone oxidoreductase subunit B11(NDUFB11) | | |  | 0.028018 | -0.3407093 |
| ENSG00000160194 | NADH:ubiquinone oxidoreductase subunit V3(NDUFV3) | | |  | 0.028846 | -0.2749307 |
| ENSG00000023287 | RB1 inducible coiled-coil 1(RB1CC1) | | |  | 2.46E-13 | -0.2403925 |
| ENSG00000070540 | WD repeat domain, phosphoinositide interacting 1(WIPI1) | | |  | 0.000293 | -0.2676249 |
| ENSG00000100219 | X-box binding protein 1(XBP1) |  |  |  | 0 | -0.3733702 |
| ENSG00000131966 | actin related protein 10(ACTR10) | |  |  | 0.005246 | -0.2959124 |
| ENSG00000118217 | activating transcription factor 6(ATF6) | | | | 1.46E-08 | -0.2183717 |
| ENSG00000064012 | caspase 8(CASP8) |  |  |  | 2.02E-06 | -0.2861564 |
| ENSG00000121691 | catalase(CAT) |  |  |  | 0.001699 | -0.2395576 |
| ENSG00000168036 | catenin beta 1(CTNNB1) |  |  |  | 0 | -0.2190651 |
| ENSG00000132912 | dynactin subunit 4(DCTN4) | |  |  | 0.000421 | -0.2107565 |
| ENSG00000233276 | glutathione peroxidase 1(GPX1) |  |  |  | 1.71E-07 | -0.1646467 |
| ENSG00000164294 | glutathione peroxidase 8 (putative)(GPX8) | |  |  | 6.00E-05 | -0.2897303 |
| ENSG00000125538 | interleukin 1 beta(IL1B) |  |  |  | 0.002734 | -1.6417523 |
| ENSG00000136244 | interleukin 6(IL6) |  |  |  | 3.42E-12 | -0.8017297 |
| ENSG00000140941 | microtubule associated protein 1 light chain 3 beta(MAP1LC3B) | | | | 0.000119 | -0.1404295 |
| ENSG00000148572 | nuclear receptor binding factor 2(NRBF2) | |  |  | 0.018546 | -0.2622648 |
| ENSG00000143106 | proteasome 20S subunit alpha 5(PSMA5) | |  |  | 0.007434 | -0.410923 |
| ENSG00000277791 | proteasome 20S subunit beta 3(PSMB3) | |  |  | 0.024462 | -0.3542541 |
| ENSG00000100804 | proteasome 20S subunit beta 5(PSMB5) | |  |  | 4.60E-14 | -0.318459 |
| ENSG00000159352 | proteasome 26S subunit ubiquitin receptor, non-ATPase 4(PSMD4) | | | | 0.000729 | -0.2279843 |
| ENSG00000103035 | proteasome 26S subunit, non-ATPase 7(PSMD7) | | |  | 0.000362 | -0.3662935 |
| ENSG00000099341 | proteasome 26S subunit, non-ATPase 8(PSMD8) | | |  | 0 | -0.1806032 |
| ENSG00000117118 | succinate dehydrogenase complex iron sulfur subunit B(SDHB) | | | | 0.012927 | -0.3189196 |
| ENSG00000143252 | succinate dehydrogenase complex subunit C(SDHC) | | |  | 2.18E-11 | -0.1963398 |
| ENSG00000145335 | synuclein alpha(SNCA) |  |  |  | 0.000649 | -0.2994271 |
| ENSG00000140740 | ubiquinol-cytochrome c reductase core protein 2(UQCRC2) | | |  | 0.011206 | -0.2930018 |
| ENSG00000233954 | ubiquinol-cytochrome c reductase hinge protein like(UQCRHL) | | | | 0.007329 | -0.5612238 |
| ENSG00000184076 | ubiquinol-cytochrome c reductase, complex III subunit X(UQCR10) | | | | 0.018227 | -0.4898016 |
| ENSG00000150991 | ubiquitin C(UBC) |  |  |  | 0.008149 | -0.3024254 |
| ENSG00000154277 | ubiquitin C-terminal hydrolase L1(UCHL1) | |  |  | 2.30E-14 | -0.2168401 |
| ENSG00000156587 | ubiquitin conjugating enzyme E2 L6(UBE2L6) | |  |  | 4.93E-08 | -0.29114 |

Table 2. Significantly downregulated DEGs (PPEE < 0.05) belonging to the enriched antigen processing and presentation pathway in fetal astrocytes cultured on CO/SPI/PCL versus CO/PCL nanofibers, analyzed via EBSeq.

| ENSEMBL GENE ID | Gene Name | | | | PPEE | Log2FC |
| --- | --- | --- | --- | --- | --- | --- |
| ENSG00000216490 | IFI30 lysosomal thiol reductase(IFI30) | |  |  | 1.61E-06 | -0.406738 |
| ENSG00000166710 | beta-2-microglobulin(B2M) | | |  | 0.03148 | -0.5666004 |
| ENSG00000135047 | cathepsin L(CTSL) |  |  |  | 0 | -0.5673933 |
| ENSG00000163131 | cathepsin S(CTSS) |  |  |  | 3.39E-06 | -0.6472505 |
| ENSG00000234745 | major histocompatibility complex, class I, B(HLA-B) | | | | 1.54E-05 | -0.3737758 |
| ENSG00000242574 | major histocompatibility complex, class II, DM beta(HLA-DMB) | | | | 0.03105 | -0.7378459 |
| ENSG00000231389 | major histocompatibility complex, class II, DP alpha 1(HLA-DPA1) | | | | 0 | -0.5482376 |
| ENSG00000204287 | major histocompatibility complex, class II, DR alpha(HLA-DRA) | | | | 0 | -1.0247279 |
| ENSG00000120837 | nuclear transcription factor Y subunit beta(NFYB) | | |  | 0.035519 | -0.3943028 |
| ENSG00000092010 | proteasome activator subunit 1(PSME1) | | |  | 1.89E-06 | -0.2305448 |
| ENSG00000100911 | proteasome activator subunit 2(PSME2) | | | | 3.81E-09 | -0.2911789 |
| ENSG00000143390 | regulatory factor X5(RFX5) | | | | 0.001237 | -0.2040137 |

Table 3. Significantly upregulated DEGs (PPEE < 0.05) belonging to the enriched focal adhesion pathway in fetal astrocytes cultured on CO/SPI/PCL versus CO/PCL nanofibers, analyzed via EBSeq.

| ENSEMBL GENE ID | Gene Name | PPEE | Log2FC |
| --- | --- | --- | --- |
| ENSG00000140992 | 3-phosphoinositide dependent protein kinase 1(PDPK1) | 9.52E-05 | 0.3950732 |
| ENSG00000105221 | AKT serine/threonine kinase 2(AKT2) | 0.039633 | 0.2594486 |
| ENSG00000174775 | HRas proto-oncogene, GTPase(HRAS) | 1.61E-05 | 0.2640539 |
| ENSG00000105976 | MET proto-oncogene, receptor tyrosine kinase(MET) | 0.029695 | 0.1195481 |
| ENSG00000136238 | Rac family small GTPase 1(RAC1) | 0.003577 | 0.1770831 |
| ENSG00000128340 | Rac family small GTPase 2(RAC2) | 1.08E-05 | 0.1658225 |
| ENSG00000134318 | Rho associated coiled-coil containing protein kinase 2(ROCK2) | 2.21E-05 | 0.3083014 |
| ENSG00000130402 | actinin alpha 4(ACTN4) | 0.015229 | 0.4160728 |
| ENSG00000162909 | calpain 2(CAPN2) | 0 | 0.2097736 |
| ENSG00000187498 | collagen type IV alpha 1 chain(COL4A1) | 0.003078 | 0.6905409 |
| ENSG00000134871 | collagen type IV alpha 2 chain(COL4A2) | 0.000419 | 0.626536 |
| ENSG00000110092 | cyclin D1(CCND1) | 1.09E-07 | 0.518548 |
| ENSG00000146648 | epidermal growth factor receptor(EGFR) | 5.86E-06 | 0.6389 |
| ENSG00000115414 | fibronectin 1(FN1) | 0.002261 | 0.6454797 |
| ENSG00000136068 | filamin B(FLNB) | 0.006964 | 0.574387 |
| ENSG00000005884 | integrin subunit alpha 3(ITGA3) | 0.000546 | 0.5849219 |
| ENSG00000082781 | integrin subunit beta 5(ITGB5) | 0.015042 | 0.5551915 |
| ENSG00000091136 | laminin subunit beta 1(LAMB1) | 1.16E-07 | 0.3253635 |
| ENSG00000172037 | laminin subunit beta 2(LAMB2) | 4.37E-05 | 0.5588246 |
| ENSG00000065534 | myosin light chain kinase(MYLK) | 3.03E-06 | 0.411884 |
| ENSG00000180370 | p21 (RAC1) activated kinase 2(PAK2) | 5.03E-06 | 0.3670292 |
| ENSG00000089159 | paxillin(PXN) | 0.032551 | 0.4565996 |
| ENSG00000143398 | phosphatidylinositol-4-phosphate 5-kinase type 1 alpha(PIP5K1A) | 0.032551 | 0.4565996 |
| ENSG00000145431 | platelet derived growth factor C(PDGFC) | 0.001226 | 0.3609575 |
| ENSG00000171914 | talin 2(TLN2) | 7.78E-05 | 0.7100066 |
| ENSG00000041982 | tenascin C(TNC) | 6.02E-05 | 0.7292577 |
| ENSG00000160293 | vav guanine nucleotide exchange factor 2(VAV2) | 0.027377 | 0.4371391 |
| ENSG00000035403 | vinculin(VCL) | 0.000237 | 0.2084969 |
